# PP2A negatively regulates the hypertrophic response by dephosphorylating HDAC2 S394 in the heart

**DOI:** 10.1038/s12276-018-0121-2

**Published:** 2018-07-26

**Authors:** Somy Yoon, Taewon Kook, Hyun-Ki Min, Duk-Hwa Kwon, Young Kuk Cho, Mira Kim, Sera Shin, Hosouk Joung, Seung Hoon Jeong, Sumin Lee, Gaeun Kang, Yunchul Park, Yong Sook Kim, Youngkeun Ahn, Julie R. McMullen, Ulrich Gergs, Joachim Neumann, Kyung Keun Kim, Jungchul Kim, Kwang-Il Nam, Young-Kook Kim, Hyun Kook, Gwang Hyeon Eom

**Affiliations:** 1 Department of Pharmacology, Chonnam National University Medical School, Hwasun, 58128 Republic of Korea; 2 Medical Research Center for Gene Regulation, Chonnam National University Medical School, Hwasun, 58128 Republic of Korea; 3 Basic Research Laboratory for Cardiac Remodeling Research Laboratory, Chonnam National University Medical School, Hwasun, 58128 Republic of Korea; 4 Department of Pediatrics, Chonnam National University Hospital, Gwangju, 61469 Republic of Korea; 5 Division of Clinical Pharmacology, Chonnam National University Hospital, Gwangju, 61469 Republic of Korea; 6 Division of Trauma Surgery, Department of Surgery, Chonnam National University Hospital, Gwangju, 61469 Republic of Korea; 7 Biomedical Research Institute, Chonnam National University Hospital, Gwangju, 61469 Republic of Korea; 8 Department of Cardiology, Chonnam National University Hospital, Gwangju, 61469 Republic of Korea; 9 Baker Heart and Diabetes Institute, Melbourne, VIC 3004 Australia; 10 Institute of Pharmacology and Toxicology, Faculty of Medicine, Martin Luther University Halle-Wittenberg, 06097 Halle, Germany; 11 Department of Anatomy, Chonnam National University Medical School, Hwasun, 58128 Republic of Korea; 12 Department of Biochemistry, Chonnam National University Medical School, Hwasun, 58128 Republic of Korea

## Abstract

Cardiac hypertrophy occurs in response to increased hemodynamic demand and can progress to heart failure. Identifying the key regulators of this process is clinically important. Though it is thought that the phosphorylation of histone deacetylase (HDAC) 2 plays a crucial role in the development of pathological cardiac hypertrophy, the detailed mechanism by which this occurs remains unclear. Here, we performed immunoprecipitation and peptide pull-down assays to characterize the functional complex of HDAC2. Protein phosphatase (PP) 2 A was confirmed as a binding partner of HDAC2. PPP2CA, the catalytic subunit of PP2A, bound to HDAC2 and prevented its phosphorylation. Transient overexpression of PPP2CA specifically regulated both the phosphorylation of HDAC2 S394 and hypertrophy-associated HDAC2 activation. HDAC2 S394 phosphorylation was increased in a dose-dependent manner by PP2A inhibitors. Hypertrophic stresses, such as phenylephrine in vitro or pressure overload in vivo, caused PPP2CA to dissociate from HDAC2. Forced expression of PPP2CA negatively regulated the hypertrophic response, but PP2A inhibitors provoked hypertrophy. Adenoviral delivery of a phosphomimic HDAC2 mutant, adenovirus HDAC2 S394E, successfully blocked the anti-hypertrophic effect of adenovirus-PPP2CA, implicating HDAC2 S394 phosphorylation as a critical event for the anti-hypertrophic response. PPP2CA transgenic mice were protected against isoproterenol-induced cardiac hypertrophy and subsequent cardiac fibrosis, whereas simultaneous expression of HDAC2 S394E in the heart did induce hypertrophy. Taken together, our results suggest that PP2A is a critical regulator of HDAC2 activity and pathological cardiac hypertrophy and is a promising target for future therapeutic interventions.

## Cardiovascular disease: A brake for heart muscle growth

A regulatory mechanism that controls how cardiac muscle responds to stress could inform development of new therapies for preventing heart failure. Physiological stimuli ranging from heavy exercise to heart attack can induce hypertrophy, an increase in cardiac muscle mass that is initially beneficial but can lead to organ failure. Researchers led by Gwang Hyeon Eom and Hyun Kook at the Chonnam National University Biomedical Research Center, Hwasungun, South Korea have found that an enzyme called protein phosphatase 2A (PP2A) keeps cardiac hypertrophy in check. PP2A binds to and inhibits a second protein known as HDAC2, which would otherwise stimulate the hypertrophic response to stress. The researchers have identified the biochemical mechanism by which PP2A inactivates HDAC2, and demonstrate that this inhibition effectively protects against hypertrophic cardiac damage in mice, revealing a possible avenue for clinical intervention.

## Introduction

The heart supplies oxygen and nutrition to the periphery by pumping blood. In certain conditions, such as hypertension, chronic exercise, myocardial infarction, and pregnancy, hemodynamic demands are elevated, and neurohormonal systems are consequently activated^1^. The increased hemodynamic demands result in a priming of the cardiac muscle to increase ventricular force to meet the demand. This series of adaptations is termed cardiac hypertrophy. The characteristic feature of cardiac hypertrophy is an increase in heart muscle mass due to the enlargement of individual cardiomyocytes without an increase in the cell population^1,2^. Although this initial hypertrophic process is considered beneficial and adaptive, sustained stresses caused by underlying disease (e.g., hypertension) can lead to irreversible pathological cardiac hypertrophy associated with cell death, fibrosis, and cardiac dysfunction. The heart can no longer contract or relax properly and will fail. Pathological hypertrophy typically progresses into irreversible heart failure^3^, and the key mechanisms responsible for this progression are not well understood. In the search for an appropriate intervention to inhibit this transition to heart failure, many research groups have studied the molecular mechanisms responsible for the development of pathological cardiac hypertrophy. Among them, one notable mechanism of cardiac hypertrophy is histone deacetylase (HDAC)-mediated cardiac remodeling.

HDACs are a group of enzymes that regulate lysine acetylation and thereby protein function^4^. Acetyl moieties do not have an ionic charge, but they can remove the cationic charge of the ammonia chain on lysine residues. In the case of histone acetylation, neutralization of the positive charge from lysine reduces the tight binding of lysine to the anionic DNA^5^, resulting in the stimulation of gene expression^6^. For this reason, histone acetyltransferases act to induce transcription, whereas HDACs function as general transcription repressors^7,8^.

To date, 18 subtypes of HDAC have been characterized into four classes: HDAC1, 2, 3, and 8 in class I; HDAC 4, 5, 6, 7, 9, and 10 in class II; seven subtypes of the sirtuin family in class III, and HDAC11 is the unique member of class IV. The functional relevance of HDACs in the development of cardiac hypertrophy has been extensively investigated^9,10^. Both class-I and class-IIa HDACs are closely associated with cardiac hypertrophy; however, their roles are quite different. Class-IIa HDACs are negative regulators of cardiac hypertrophy. They act either by interfering with the binding of myocyte enhancer factor 2c or by direct suppression of gene expression of pro-hypertrophic molecules^11^. Class-IIa HDACs undergo phosphorylation by either protein kinase D or calcium/calmodulin-dependent protein kinase II, which causes cytoplasmic redistribution by the 14-3-3 protein^12,13^. In contrast, among class-I HDACs, HDAC2 mainly plays a positive role in cardiac hypertrophy^14^. Global deletion of HDAC2 allows resistance against hypertrophic stimuli^15^, whereas cardiac-specific overexpression of HDAC2 results in the development of cardiac hypertrophy^16^. The phosphorylation state of HDAC2 regulates its activity, and hypertrophic stress induces its phosphorylation. α- or β-adrenergic agonists, neurohormonal agonists, pressure overload, or other hypertrophic stresses activate casein kinase (CK) 2α1^16^ and p300/CBP-associated factor (pCAF)^17^. pCAF acetylates HDAC2 K75 and then induces CK2α1-mediated phosphorylation of HDAC2 at serine 394. This mechanism suggests that lysine 75 acetylation is required for S394 phosphorylation^17^.

In addition to HDAC2 S394, which is a hypertrophic stress-responsive phosphorylation target, other phosphorylation sites are involved in enzyme activity: S394, S422, and S424^18^. The basal activity of HDAC2, the repression of histone acetylation, requires the simultaneous phosphorylation of both S422 and S424^18^. Alternatively, the phosphorylation of S394 in response to exogenous stimuli provokes the hypertrophic response. Although we have reported that S394 phosphorylation and its kinase activity is directly associated with cardiac hypertrophy^16^, previous reports have yet to elucidate in detail the mechanisms used to maintain the phosphorylation balance.

Here, we propose a novel mechanism for the regulation of HDAC2 phosphorylation status and its activity in the development of cardiac hypertrophy. The physical interaction of the protein phosphatase (PP) 2 A with HDAC2 results in the dephosphorylation and inactivation of HDAC2. Thus, we propose that changing the HDAC2 binding partner and the subsequent regulation of its activity are critical in the development of hypertrophic phenotypes.

## Materials and methods

### Reagents

Isoproterenol, phenylephrine, bovine serum albumin (BSA), and 2,2,2-tribromoethanol were purchased from Sigma (Sigma-Aldrich Corp, St. Louis, MO, USA). Okadaic acid was purchased from Abcam (Abcam, Cambridge, UK). LB-100 was purchased from Selleckchem (Selleckchem, Houston, TX, USA).

The following antibodies were used at the dilution listed: HDAC2 (ab12169, 1:5000), pHDAC2 pS394 (ab75602, 1:1000), PPP2CA (ab33537, 1:2000), and CK2α1 (ab70774, 1:5000) from Abcam (Abcam); sarcomeric α-actinin (for immunocytochemistry, A7811, 1:500) from Sigma (Sigma-Aldrich Corp.); rabbit polyclonal anti-HDAC2 (51-5100, 1:1000) and anti-V5 (R96025, 1:2000) from Invitrogen (Invitrogen Corp., Waltham, MA, USA); anti-HA (11 583 816 001, 3 μg/blot) from Roche (Hoffmann-La Roche, Basel, Switzerland); anti-Myc from Cell Signaling (#2278, 1:1000, Cell Signaling Technology, Danvers, MA, USA) and Santa Cruz (sc-40, 1:1000, Santa Cruz Biotechnology Inc., Santa Cruz, CA, USA); normal mouse IgG (sc-2025) and normal rabbit IgG (sc-2027) from Santa Cruz (Santa Cruz Biotechnology Inc.); HRP-conjugated secondary antibody against mouse IgG (#7076) or rabbit IgG (#7074) from Cell Signaling (Cell Signaling Technology); Alexa Fluor 568-conjugated anti-rabbit IgG (A11011, 1:500) and Alexa Fluor 568-conjugated anti-mouse IgG (A11004, 1:500) from Molecular Probes (Molecular Probes, Eugene, OR, USA); and rabbit polyclonal anti-phosphor-422/phosphor-424 HDAC2 antibody (1:1000) was generated by a commercial company (Peptron, Daejeon, Korea). The epitope sequence for immunization was as follows: IACDEEFS^p^DS^p^EDEGEGG. The antibody specificity was confirmed using a HDAC2 S422A/S424A mutant, which is presented in Supplementary Figure 1.

### Plasmid and siRNA

Various mutants of HDAC2 such as *pcDNA3.1-HDAC2 S394A, S394E*, and *S422/424A-V5* were generated by site-directed mutagenesis (Agilent Technologies, Santa Clara, CA, USA) from *pcDNA3.1-HDAC2-WT-V5. pcDNA6-3xHA-CK2a1* was subcloned from *PGS5-CK2a1-HA. pcDNA6-3xHA-PPP2CA* was subcloned from *pcDNA6-PPP2CA-myc*. All plasmids were verified by direct DNA sequencing.

### Cell culture

H9c2 was originally obtained from the American Type Culture Collection (ATCC, Manassas, VA, USA). Cells were maintained in Dulbecco’s modified Eagle’s medium (DMEM, Thermo Inc., Waltham, MA, USA) supplemented with 10% fetal bovine serum (Thermo Inc.) and 1% antibiotics (penicillin and streptomycin) (Thermo Inc.) at 37 °C with 5% CO_2_/95% air. Cell passaging was performed every 2 or 3 days to prevent cells from becoming confluent.

### Primary cell culture

Neonatal rat ventricular cardiomyocytes (NRVCs) were harvested from both ventricles of 1- to 2-day-old Sprague Dawley rat hearts. The ventricles were chopped into small pieces, and collagen digestion was performed in 0.1% type-II collagenase in ADS buffer (20 mmol/L HEPES, pH 7.4, 120 mmol/L NaCl, 5.5 mmol/L glucose, 11 mmol/L NaH_2_PO_4_, 5.4 mmol/L KCl, and 0.44 mmol/L MgSO_4_ in distilled water) for 30 min at 37 °C with gentle agitation. By adding growth medium (DMEM high glucose supplemented with 10% fetal bovine serum and 1% antibiotics), enzyme digestion was completed. Cells were collected by a brief centrifugation at 1000 RCF for 10 min. Then, the supernatant was removed, and the pellet was resuspended in growth medium. The cells were pre-plated for 1 h to remove fibroblasts. After counting, the cells were plated in culture dishes coated with 0.2% gelatin.

### Animal models

For measurements of in vivo hypertrophy, either the beta-adrenergic agonist model or the pressure- overload model was applied. An Alzet Osmotic Pump (Durect Corp., Cupertino, CA, USA) containing 30 mg/kg/day isoproterenol was dissolved in 100 μL of vehicle (0.9% NaCl, pH 4.0) and implanted under the dorsal skin of mice under anesthesia with 2,2,2-tribromoethanol. For the pressure-overload model, the ascending aorta was partially constricted. After anesthesia with 2,2,2-tribromoethanol, artificial ventilation was maintained. The mediastinum was exposed by median sternotomy. The ascending aorta was visualized after careful removal of the pericardium. Aortic banding was induced by use of 7-0 silk suture. Five days after aortic banding, an echocardiogram was performed to measure the constricted lumen. The inclusion criterion for aortic banding was greater than 70% constriction. Cardiac hypertrophy was induced for 14 days. Hypertrophy was assessed by the heart weight per body weight ratio or the heart weight per tibia length ratio. Animal experimental procedures followed the guidelines of the National Institutes of Health. All in vivo experiments were approved by the Chonnam National University Medical School Research Institutional Animal Care and Use Committee (CNU IACUC-H-20174-29).

### Adenovirus

Adenoviruses expressing HDAC2 wild-type, HDAC2 S394A, or HDAC2 S394E were generated by using the Ad-Easy system (Agilent Technologies). PPP2CA-HA adenovirus was purchased from Abm (Viking PI, Richmond, Canada). Infection and expression of HDAC2 mutant viruses in the cells were checked by a GFP reporter driven by the IRES system. Ad-PPP2CA-HA expression was confirmed by western blot analysis with an antibody against the HA epitope.

### In vivo delivery of adenovirus

For transient overexpression of PPP2CA in the heart, adenovirus expressing PPP2CA-HA was utilized. In total, 8-week-old male CD1 mice were purchased (Orient Bio., Seongnam, Korea). Cardiac hypertrophy was induced by 30 mg/kg/day isoproterenol as described above. The day after minimally invasive surgery for implantation of the osmotic pump, 1 × 10^8^ IFU Ad-PPP2CA-HA virus was diluted in 100 μL of sterile saline and injected via the tail vein. An equal IFU of Ad-GFP was delivered for the negative control group. Infection of Ad-PPP2CA-HA was confirmed both by quantitative real-time PCR in heart tissue and western blotting with an anti-HA antibody.

### Immunoprecipitations and western blots

NRVCs and H9c2 were washed with ice-cold PBS and collected by brief centrifugation. Lysates were obtained by addition of 1% NP-40 (Igepal CA-630, 50 mmol/L Tris-HCl, pH 8.0, 150 mmol/L NaCl, 5 mmol/L EDTA, 2 mmol/L NaF, and protease inhibitor mixture [Calbiochem, La Jolla, CA, USA]). The lysate was briefly sonicated and precipitated by centrifugation (14,000 RCF) for 15 min. The aqueous layer was utilized for western blotting or immunoprecipitation. To obtain lysates from in vivo hypertrophy mouse models, both atria and large vessels were removed from the heart. The left ventricular free wall was then isolated from the heart and chopped into small pieces. Tissues were lysed by addition of 1% NP-40 buffer before homogenization and sonication. The lysate was precipitated by centrifugation (14,000 RCF) for 15 min.

For immunoprecipitation, ~1–2 mg of lysate was incubated with the primary antibody at 4 °C overnight with continuous rotation (1 μg of antibody per 1 mg of lysate). The protein–antibody complex was then captured using Protein G Plus agarose beads (Santa Cruz Biotechnology Inc.). After 2 h of rotation, bead complexes were precipitated by centrifugation and washed twice with lysis buffer. The denaturation and reducing process was performed by boiling for 5–7 minutes after mixing with NuPAGE SDS sample buffer (Invitrogen Corp.) containing β-mercaptoethanol. After separation by SDS-PAGE, the protein was transferred to PVDF membranes (Merck Millipore Corp., Darmstadt, Germany). Probed target proteins were visualized using the HRP substrate (ECL, Merck Millipore Corp.).

### Quantitative real-time polymerase chain reaction (real-time PCR)

RNA was extracted from cells or tissues using a QIAzol Kit (Qiagen, Hilden, Germany). cDNA was synthesized using a commercially available kit (Invitrogen Corp.). Total RNA was incubated with random hexamers and a dNTP mix at 70 °C for 10 min. After incubation, M-MLV, RNase inhibitor, 0.1 mmol/L DTT, and 5 × FS buffer were added according to the manufacturer’s instructions. The samples were then incubated at 42 °C for 1 h. The cDNA synthesis was terminated at 95 °C for 5 min. Reaction mixture aliquots were used as templates for PCR. Amplification reactions were performed using a DNA thermal cycler (MasterCycler; Eppendorf, Hamburg, Germany). Quantitative real-time PCR analysis was performed in triplicate with a Rotor-Gene Q (Qiagen) using TOPreal™ qPCR 2 × PreMIX (Enzynomics, Daejeon, Korea). The amounts of mRNAs were normalized to the 18 S rRNA endogenous control. Oligo sets for Nppa and Nppb were purchased (Bioneer, Daejeon, Korea). The specific sequences for PCR were as follows:

18S rRNA, sense: 5ʹ-GTAACCCGTTGAACCCCATT-3ʹ.

18S rRNA, antisense: 5ʹ-CCATCCAATCGGTAGTAGCG-3ʹ.

PPP2CA, sense: 5ʹ-GATCACACAAGTTTATGGTTTCTATGATGAA-3ʹ.

PPP2CA, antisense: 5ʹ-TATGATCCAGTGTATCTATAGATGGCGAG-3ʹ.

### Luciferase and β-galactosidase assay

Luciferase activity was measured to check *Nppa* expression using a commercially available kit (GloMax^®^; Promega, Madison, WI, USA) as per the manufacturer’s guidelines. Plasmids containing *Nppa*-luciferase and β-galactosidase were transfected into H9c2 cells or cardiomyocytes. Two days after transfection, cells were washed with PBS and then lysed in 100 μL of 5 × reporter lysis buffer with vigorous agitation. After confirmation of lysis, the lysate was mixed with luciferase assay reagent, and promoter activity was measured with a luminometer. β-galactosidase expression was determined using a MAXLINE (Promega) microplate reader after reaction with its substrate. The β-galactosidase assay utilized an internal control to adjust for transfection efficiency.

### Histone deacetylase (HDAC) activity assay

HDAC activity was measured using a commercial kit (HDAC-Glo^TM^ I/II Assays; Promega). Protein lysates were prepared with 1% NP buffer excluding either EDTA or EGTA to avoid zinc chelation. After immunoprecipitation with anti-HDAC2, the bead-immunocomplex was mixed with HDAC assay substrate and incubated for more than 15 min at room temperature. HDAC activity was detected using a luminometer. The nonspecific IgG-precipitated HDAC activity was regarded as the blank and was subtracted from the basal level.

### L-Homopropargylglycine incorporation assay

De novo protein synthesis was measured using Click-it^®^ HPG Alexa Fluor^®^ Protein Synthesis Assay Kits (Thermo Inc., Waltham, MA, USA). Appropriately prepared NRVCs were incubated overnight and washed with PBS after aspiration of conditioned media. Further synthesis of protein proceeded with l-homopropargylglycine (HPG)-containing media for 30 min. Incorporated amino acids were targeted by Alexa Fluor 594 azide. Red fluorescence was measured using fluorescence microscopy and quantified using the NIS-Elements AR program (Nikon Inc, Tokyo, Japan). The mean intensity of each cell was calculated as the amount of protein synthesis after subtraction of the absorbance of empty space.

### Immunocytochemistry and cell size measurement

Conditioned NRVCs were fixed with 3.7% paraformaldehyde (Sigma-Aldrich Corp.) for 10 min, and cells were then washed with PBS containing 0.5% BSA (0.5% BSA/PBS). After blocking with 3% normal goat serum (Thermo Inc.) at room temperature for 30 min, the cells were incubated overnight with primary antibodies (1:500) in permeabilization buffer (0.2% Triton X-100, 1% BSA, and PBS). Primary antibodies against sarcomeric alpha-actinin were probed again using Alexa 568-conjugated secondary antibodies (anti-mouse; Molecular Probes). An antifade solution containing 6-diamidino-2-phenylindole (DAPI, Molecular Probes) was added for nuclear staining. Chamber slides were covered with mounting slides. Cell sizes were measured using the NIS-Elements AT program (Nikon Inc.). More than 100 cells from individual experimental sets were measured.

### Genetically engineered mice

Ppp2ca was expressed by the cardiac-specific promoter myosin heavy chain 6^19^. The genotype of each mouse was confirmed by PCR with its specific primer. The sequence information is as follows: Ppp2ca, sense: 5ʹ-CCCTTACCCCACATAGACC-3ʹ.

Ppp2ca, antisense: 5ʹ-CTTAAACACTCGTCGTAGAACC-3ʹ.

### Cross-sectional area measurement

To evaluate in vivo hypertrophy, the cross-sectional area of cardiac muscle fibers was measured. Each heart was fixed with 3.7% paraformaldehyde, and Masson’s trichrome staining was performed. Horizontally sectioned myotubes in the left ventricular free wall were used for the calculation. The circumference of the muscle fiber was measured using automated software (NIS-Elements AT program; Nikon Inc.). If the longest diameter was more than twofold greater than the shortest diameter, the fibers were excluded because the myotube area was considered to be a longitudinal or oblique section.

### Densitometer analysis

The alteration of binding between HDAC2 and PPP2CA by agonist stimuli or by forced expression was quantified using ImageJ software. The output results were divided by their input densities, which were divided again by their negative control. The results are presented as the fold change in parallel with representative western blot images in Fig. 3a–d.

### Quantification and statistics

Each experiment was confirmed by iteration of more than three independent sets. To acquire the pooling data and quantification, whole raw data were divided by mean data acquired from a negative control group. The negative control was regarded as 1, and the fold changes in each experimental group were obtained. Statistical analyses were performed with PASW Statistics 23 (SPSS, IBM Corp, Chicago, IL, USA). Outliers were determined by Grubb’s test and excluded. To analyze more than three groups, one-way analysis of variance was used. Tukey’s honestly significant difference was utilized for post hoc tests. When Levene’s test for measurement of equal variance was not satisfied, Dunnett’s T3 test was utilized instead of a post hoc test. *p* values <0.05 were considered statistically significant.

## Results

### PP2A as a functional complex for HDAC2

Previously, we have clearly demonstrated that HDAC2 S394 phosphorylation and the resultant enzymatic activation are indispensable for the development of cardiac hypertrophy^16,17^. These observations further raised the fundamental questions of the identity of the phospho-specific HDAC2 binding partners and how they affect HDAC2 activity in response to hypertrophic stimuli. To answer this question, we first attempted to identify the phosphatase responsible for the dephosphorylation of HDAC2.

**Fig. 1:**
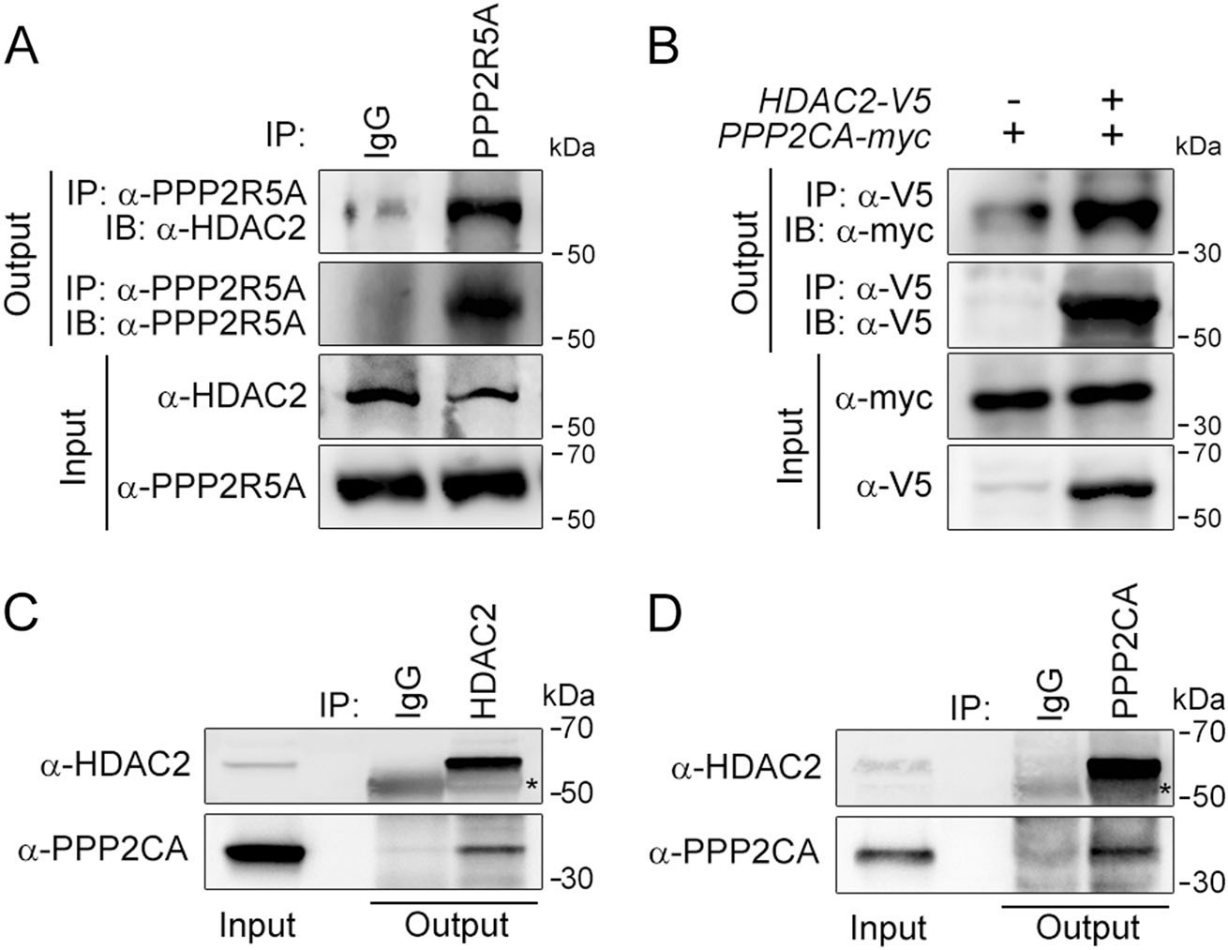
HDAC2 physically interacts with PP2A. **a** To confirm a physical interaction between PPP2R5A, the regulatory subunit of PP2A, and HDAC2 in NRVCs, primary cultured cardiomyocytes, 2 mg of protein was immunoprecipitated with an anti-PPP2R5A antibody, and the HDAC2 interaction was visualized with an anti-HDAC2 antibody. **b–d** The catalytic subunit of PP2A, PPP2CA, also bound to HDAC2. To check the interaction in H9c2 cells, a cardiomyoblast cell line, *HDAC2-V5* and *PPP2CA-myc* were transiently transfected, and cells were lysed with 1% NP-40 lysis buffer. Immunoprecipitation was performed with anti-V5 antibody followed by immunoblotting with anti-Myc antibody. **c** To check the endogenous interaction in NRVCs, primary cultured cardiomyocytes were lysed in 1% NP-40 lysis buffer. Immunoprecipitation was performed with anti-HDAC2 antibody followed by immunoblotting with anti-PPP2CA antibody. **d** PPP2CA physically interacted with HDAC2 in mouse heart. Two milligrams of heart lysates were immunoprecipitated with anti-PPP2CA. HDAC2 successfully precipitated with PPP2CA

A previous report showed that a PP2A inhibitor can increase HDAC2 phosphorylation^20,21^. The major serine/threonine phosphatase PP2A is ubiquitously expressed^19,22–24^. PP2A is a holoenzyme that consists of three subunits: the B subunit determines substrates, the C subunit functions as a phosphatase, and the A subunit serves as a scaffold. We first tested whether the B subunit of PP2A, also known as the regulatory subunit, bound to HDAC2 in the heart. The immunoprecipitation assay revealed a physical interaction between HDAC2 and PPP2R5A in primary cultured myocytes (neonatal rat ventricular cardiomyocytes, NRVCs) (Fig. 1a). Because PPP2CA is a catalytic subunit of PP2A that is linked to PPP2R5A, we checked whether PPP2CA also physically interacted with HDAC2. Physical binding of HDAC2 to PPP2CA was observed in H9c2 cells (Fig. 1b). The endogenous interaction between HDAC2 and PPP2CA was also verified in primary cultured NRVCs with the corresponding antibodies (Fig. 1c, d).

### Negative regulation of phosphorylation and HDAC2 enzyme activity by PPP2CA

**Fig. 2:**
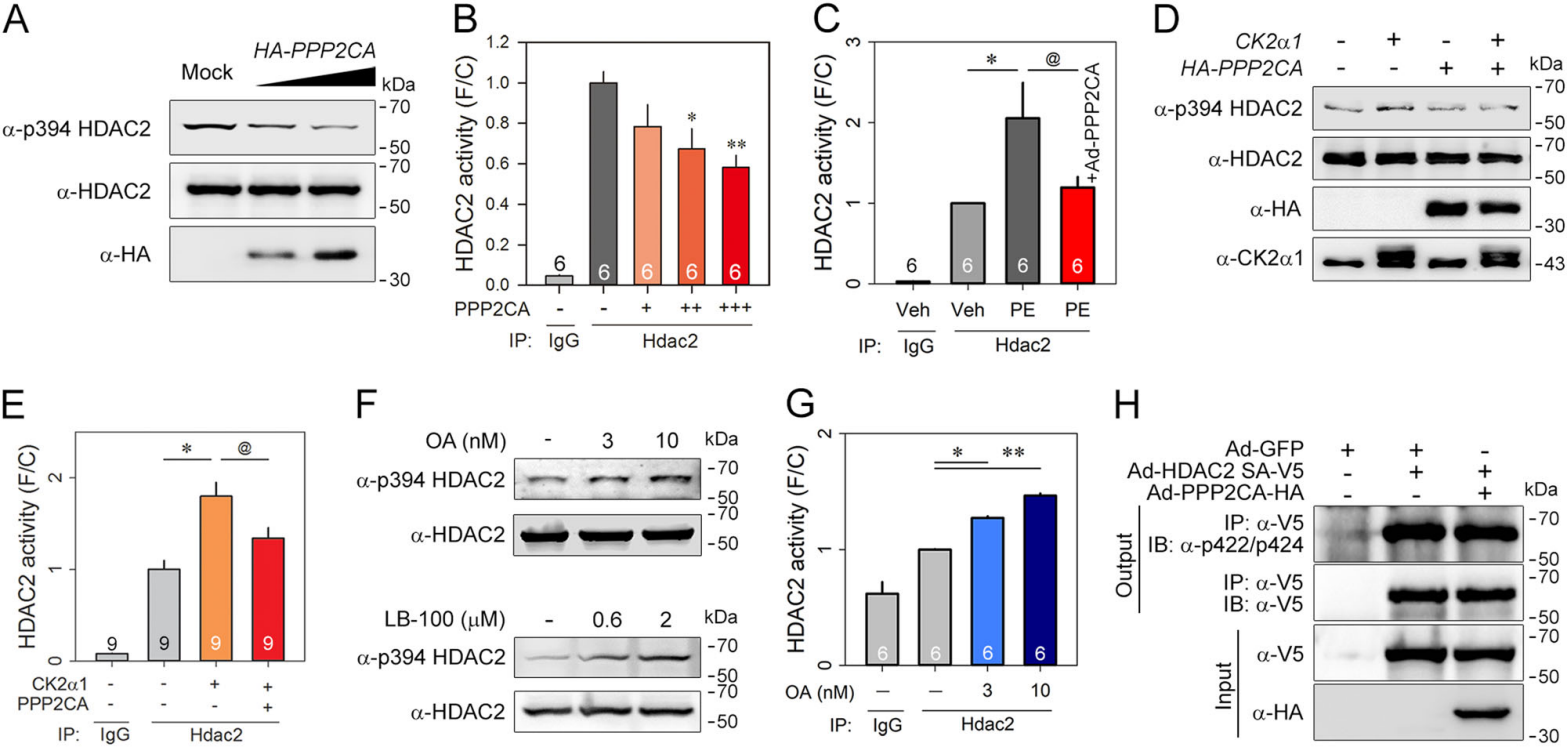
PPP2CA specifically regulates both HDAC2 phosphorylation and subsequent activation. **a** PPP2CA reduced HDAC2 S394 phosphorylation. HDAC2 phosphorylation was decreased in a PPP2CA-HA dose-dependent manner in H9c2 cells, a cardiomyoblast cell line. **b** The intrinsic activity of HDAC2 was decreased in a PPP2CA dose-dependent fashion. **c** Adenovirus expressing PPP2CA (Ad-PPP2CA) abridged HDAC2 hyperactivation induced by treatment with 20 μmol/L phenylephrine (PE), an alpha-adrenergic agonist, in NRVCs. PE-induced enzymatic activation of HDAC2 was significantly diminished by simultaneous infection of PPP2CA. The changes were displayed as the fold change by dividing by the mean value of the control group. PPP2CA attenuated HDAC2 phosphorylation and thereby activation induced by CK2α1. **d**, **e** CK2α1-induced HDAC2 phosphorylation (**d**, 2nd lane) and HDAC2 activation (**e**, 2nd bar) were completely blocked by co-transfection of PPP2CA (**e**, 4th lane, and **e**, 4th bar) in H9c2 cells. Okadaic acid (OA), a selective PP2A inhibitor, induced an increase in phosphorylation (**f**, upper panel) and intrinsic activity (**g**) of HDAC2. LB-100, an alternate PP2A inhibitor, also induced HDAC2 S394 phosphorylation (**f**, lower panel). **h** PPP2CA specifically regulated S394 phosphorylation of HDAC2. PPP2CA failed to reduce phosphorylation of S422 and S424 phosphorylation in the HDAC2 S394A mutant. The designated number in (**b**) and (**c**) indicated an independent experimental set. Data are presented as the mean ± SEM. * and @ indicate *p* < 0.05. In case of **p < 0.01

To assess the functional significance of the binding of PPP2CA, S394 phosphorylation of HDAC2 was checked after forced expression of PPP2CA. HDAC2 phosphorylation was significantly decreased by PPP2CA in a dose-dependent manner (Fig. 2a). We postulated that PPP2CA would negatively regulate phospho-dependent HDAC2 activity. To understand the functional relevance of PPP2CA for HDAC2 activity, we transfected H9c2 cells with PPP2CA and then measured HDAC2 activity. The basal activity of HDAC2 was decreased in a PPP2CA dose-dependent manner (Fig. 2b). Next, NRVCs were treated with PE and then exposed to an adenovirus-expressing PPP2CA-HA (Ad-PPP2CA). PE successfully increased HDAC2 activity, which was attenuated by simultaneous expression of PPP2CA (Fig. 2c). We next investigated whether PPP2CA could reduce the S394 phosphorylation induced by HDAC2 kinase. HDAC2 phosphorylation was selectively increased by CK2α1, which was completely abolished when PPP2CA was simultaneously expressed (Fig. 2d). Similarly, PPP2CA significantly blocked the CK2α1-induced activation of HDAC2 (Fig. 2e). To confirm the effect of PPP2CA on HDAC2 phosphorylation, the selective PP2A inhibitors, okadaic acid (OA)^25^ and LB-100^26,27^, were tested. Both HDAC2 phosphorylation (Fig. 2f) and activation (Fig. 2g) were significantly increased in a dose-dependent manner. Because PPP2CA blocked the hypertrophic stress-induced phosphorylation and activation of HDAC2, we concluded that PPP2CA functions as a phosphatase in association with cardiac hypertrophy.

Three serine residues in HDAC2 are known as targets for HDAC phosphorylation: S394, S422, and S424. Both S422 and S424 are basally phosphorylated and are important for basal transcription-repressing activity when HDAC2 forms a complex together with mSin3A, CoREST, or NuRD^18,28–30^. In contrast, S394 phosphorylation is inducible in response to exogenous signals^16^. We investigated whether PPP2CA affected basal phosphorylation. First, we generated a phospho-422/phospho-424 HDAC2 antibody and checked the specificity utilizing an HDAC2 S422A/S424A mutant. The phospho-422/phospho-424 antibody failed to recognize HDAC2 S422A/S424A protein (Supplementary Figure 1). To confirm the absence of PPP2CA-activity on phosphorylation at sites other than S394, we checked phosphorylation status by utilizing HDAC2 S394A. Immunoblots probed with the phospho-422/phospho-424 antibody revealed that PPP2CA failed to reduce phosphorylation at those serine residues (Fig. 2h). Thus, we concluded that PPP2CA does not affect the basal phosphorylation of S422/S424, which led us to focus on inducible S394 phosphorylation.

### Dissociation of PPP2CA from HDAC2 in response to hypertrophic stimuli

We have previously demonstrated that hypertrophic stresses induce HDAC2 S394 phosphorylation^16^. In contrast, in the present work, PPP2CA reduced HDAC2 S394 phosphorylation (Fig. 2). This finding led us to question the regulatory role of PPP2CA on HDAC2 phosphorylation in the hypertrophic condition. First, we checked whether the interaction of PPP2CA with HDAC2 was affected by hypertrophic stresses.

**Fig. 3:**
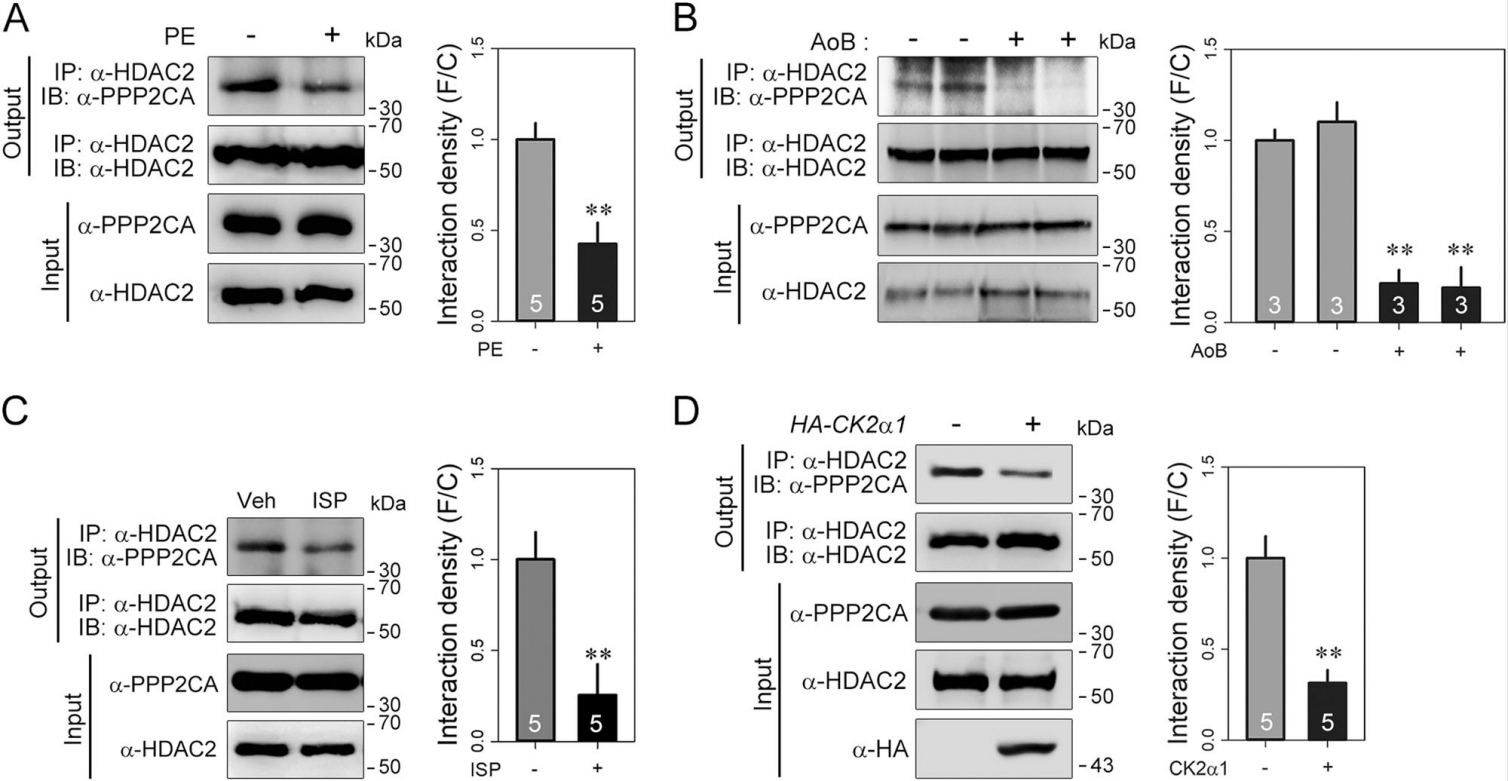
PPP2CA dissociates from HDAC2 in response to hypertrophic stimuli. The binding of PPP2CA to HDAC2 was reduced by hypertrophic stresses (**a–c**). In cardiomyocytes stimulated with 20 μmol/L PE, the interaction between HDAC2 and PPP2CA was decreased (**a**). Cardiac hypertrophy induced either by aortic banding (AoB) (**b**) or by isoproterenol (ISP, 30 mg/kg/day), a beta-adrenergic agonist, also provoked the dissociation of PPP2CA from HDAC2 (**c**). **d** To assess whether the hypertrophic stress-induced dissociation is HDAC2 phosphorylation-dependent CK2α1, an HDAC2 kinase was added to the immunoprecipitation assay. HDAC2 phosphorylation also detached PPP2CA from HDAC2. Data are presented as the mean ± SEM. **p < 0.01. White numbers in each bar denote the independent set used for densitometer analysis

The physical interaction between HDAC2 and PPP2CA was decreased by phenylephrine (PE) in cardiomyocytes (Fig. 3a). For an in vivo hypertrophy model, we utilized two experimental models: aortic banding (AoB) and infusion of isoproterenol (ISP) in mice with a micro-osmotic pump. As in the in vitro experiment, PPP2CA dissociated from HDAC2 in the heart tissues obtained either from AoB mice (Fig. 3b) or ISP-stimulated mice (Fig. 3c). In addition to hypertrophic signaling, PE and ISP can affect many other signaling cascades in cells. Therefore, based on our previous report^16^ showing that CK2α1 specifically induces the phosphorylation of HDAC2 S394, we attempted to check whether forced phosphorylation by CK2α1 also affected the interaction between PPP2CA and HDAC2. The binding of PPP2CA to HDAC2 was reduced by CK2α1 (Fig. 3d). In summary, hypertrophic signals induce the dissociation of PPP2CA from HDAC2, which is then followed by CK2α1-induced phosphorylation of HDAC2.

### Regulation of the hypertrophic response by PP2A

**Fig. 4:**
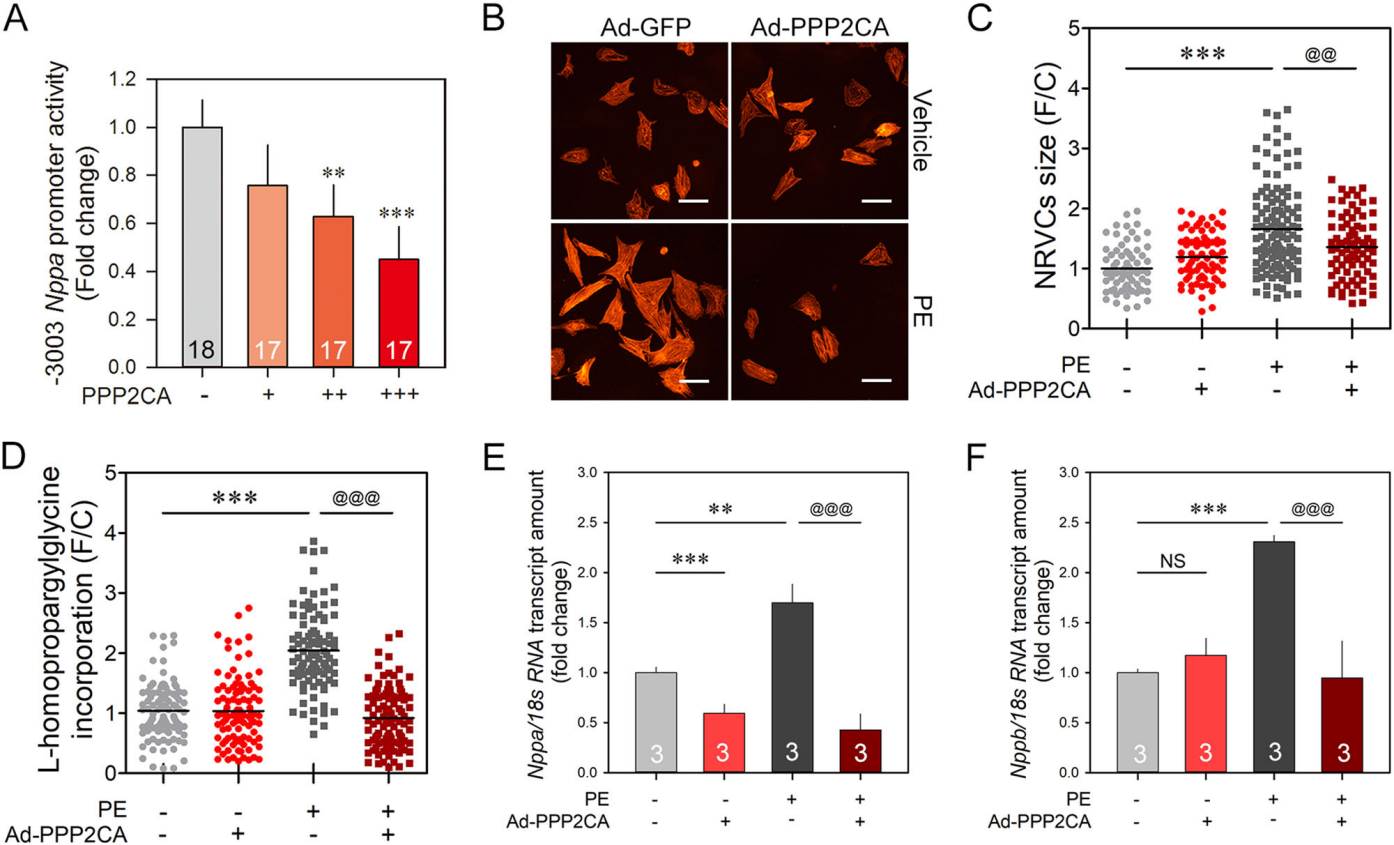
PPP2CA negatively regulates hypertrophic responses. **a** –3003 *Nppa* reporter activity in H9c2 cells was decreased in a PPP2CA dose-dependent manner. **b**, **c** Cell size measurement in NRVCs. Cardiomyocyte-specific sarcomeric alpha-actinin was visualized by immunocytochemistry analysis. Treatment with 20 μmol/L PE induced an increase in the cell surface area, a hallmark of cardiomyocyte hypertrophy. Ad-PPP2CA abolished the PE-induced cell size enlargement. The dots depict individual cell size. **d** De novo protein synthesis was evaluated by quantification of the incorporation of l-homopropargylglycine (HPG) in NRVCs. NRVCs were infected with Ad-PPP2CA for 48 h and exposed to 20 μmol/L PE. Cells were cultured in HPG-containing methionine-free medium for 1 h, and then the incorporated HPG was ligated to Alexa Fluor 594 azide for visualization. The signal density of the cells was determined by fluorescence microscopy and then quantified using software. Ad-PPP2CA significantly attenuated de novo protein synthesis driven by PE. **e**, **f** PE-induced early hypertrophic marker gene expression was regulated by Ad-PPP2CA. Both Nppa (**e**) and Nppb (**f**) were successfully increased by PE stimuli, which was blocked by infection of Ad-PPP2CA. Numbers in the bars (**a**, **e**, **f**) indicate the experimental set. White scale bars indicate 15 μm. @@ indicates *p* < 0.01. *** and @@@ depict *p* < 0.001

Having shown that PPP2CA regulates both the phosphorylation and the intrinsic activity of HDAC2, we hypothesized that PPP2CA might negatively regulate the hypertrophic response. To obtain direct evidence of the anti-hypertrophic property of PPP2CA, we first checked the promoter activity of natriuretic peptide precursor A (*Nppa*) encoding an atrial natriuretic factor (ANP). PPP2CA decreased *Nppa*-luciferase activity in a dose-dependent manner (Fig. 4a). Next, we measured the changes in individual cardiomyocyte size after stimulation with a hypertrophic agonist with or without PPP2CA. PPP2CA itself did not alter the cell size of NRVCs, but it successfully blocked PE-induced cardiomyocyte hypertrophy (Fig. 4b, c, and Supplementary Figure 2).

It is widely accepted that de novo protein synthesis is one of the most reliable markers to evaluate cardiac hypertrophy^31^. To investigate whether PPP2CA negatively regulated cardiac hypertrophy, we performed a radioisotope-free experiment using L-homopropargylglycine (HPG), a methionine analog that is incorporated into protein during de novo synthesis. Incorporated HPG was visualized by fluorescence microscopy and quantified. The amount of HPG in PE-stimulated NRVCs was twofold that in vehicle-treated NRVCs. Similar to the anti-hypertrophic function shown in Fig. 4b, c, Ad-PPP2CA significantly reduced protein synthesis, which was potentiated by PE (Fig. 4d).

The natriuretic peptide family is a well-known early hypertrophic marker. We tested whether Ad-PPP2CA was also able to regulate hypertrophy-associated gene expression. Phenylephrine (20 µmol/L) significantly induced both Nppa and Nppb in NRVCs, and this induction was completely blocked by infection with adenovirus-expressing PPP2CA (Fig. 4e, f).

### Exaggeration of the hypertrophic response by PP2A inhibitors

**Fig. 5:**
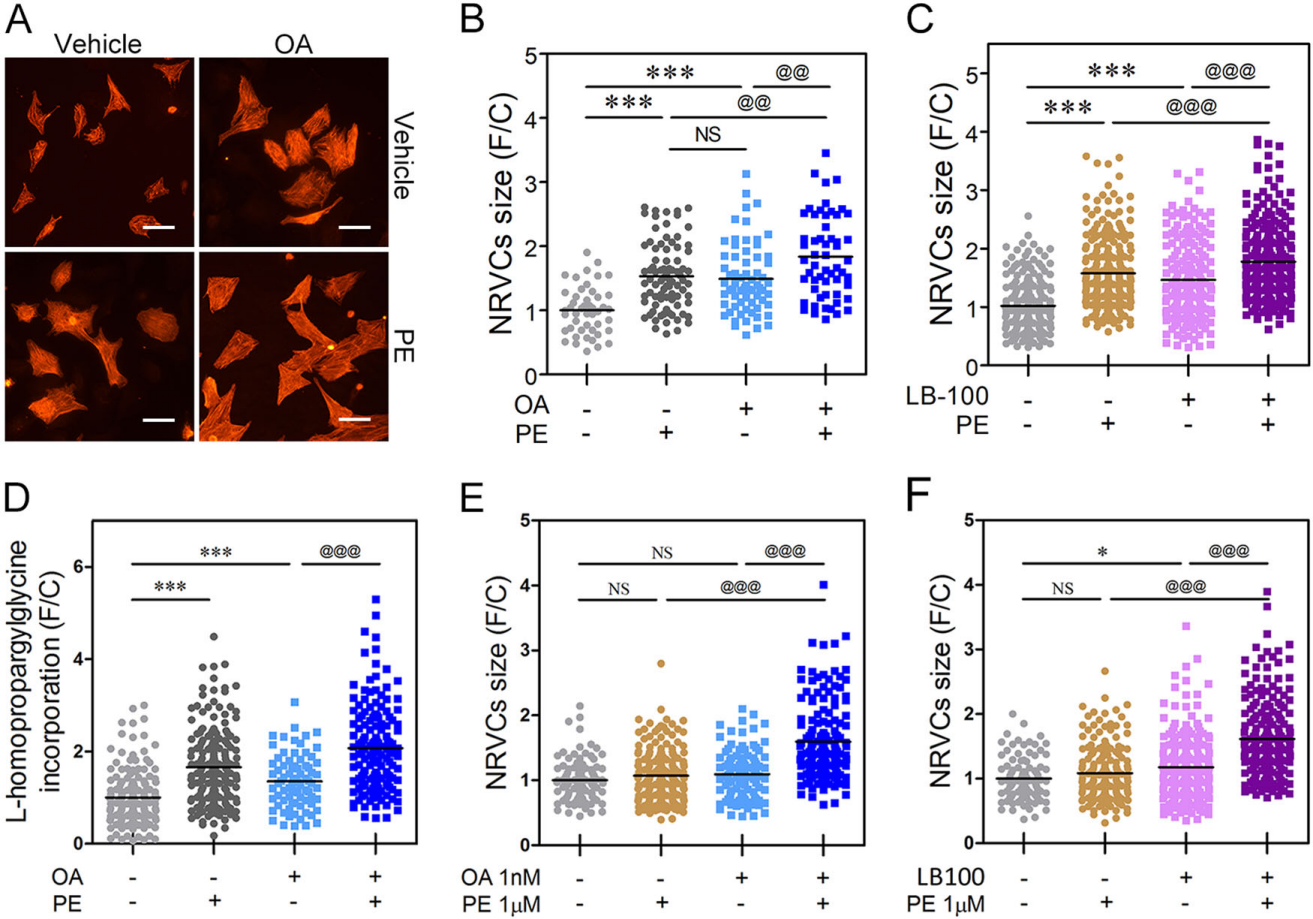
PP2A inhibitor augments the hypertrophic response. **a–d** PP2A selective inhibitors successfully induced cardiomyocyte hypertrophy. **a**, **b** OA itself significantly induced cardiomyocyte enlargement, which was further potentiated by 20 μmol/L PE. **c** LB-100, an alternative PP2A inhibitor, also provoked cardiomyocyte hypertrophy, and this effect was further exaggerated by 20 μmol/L PE. **d** Measurement of de novo protein synthesis by use of the l-homopropargylglycine (HPG) assay. The PP2A selective inhibitor, OA, induced cardiomyocyte hypertrophy, which was further potentiated by exposure to 20 μmol/L phenylephrine (PE). **e**, **f** A low dose of PE, which failed to induce hypertrophy itself, also exaggerated the PP2A inhibitor effects. OA or LB-100 provoked cardiomyocyte hypertrophy, and those effects were further exaggerated by 1 μmol/L PE, although the low dose of PE itself could not induce hypertrophy. Every dot implicates data acquired from an individual cardiomyocyte. * indicates *p* < 0.05. @@ means *p* < 0.01. *** and @@@ depict *p* < 0.001. NS not significant

Because both OA and LB-100 themselves specifically induce HDAC2 hyperphosphorylation, we measured the hypertrophic phenotype of NRVCs in the presence of those inhibitors. OA (Fig. 5a, b, and Supplementary Figure 3) or LB-100 (Fig. 5c) successfully induced cardiomyocyte enlargement even without any hypertrophic stimuli. Like the cell size measurement result, HPG incorporation was increased in the presence of OA (Fig. 5d). This PP2A inhibitor-induced cardiomyocyte hypertrophy was significantly potentiated by phenylephrine (Fig. 5a–d). The early hypertrophic genes *Nppa* and *Nppb* were also significantly increased by PP2A inhibitors (Supplementary Figure 4).

We next tested whether a low concentration of a hypertrophic agonist, which is not sufficient to induce hypertrophy, also exaggerated PP2A inhibitor-induced hypertrophy. Cardiomyocytes were significantly enlarged by adding PP2A inhibitors with a low dose of PE (Fig. 5e, f). Thus, we concluded that PPP2CA blocks the hypertrophic response.

### Regulation of cardiomyocyte hypertrophy by PP2A via HDAC2 S394 dephosphorylation

PP2A has numerous substrates in the cell. Hence, we questioned whether the PPP2CA-induced anti-hypertrophic mechanism is dependent on HDAC2 S394 phosphorylation. To assess this phenomenon, we first checked the *Nppa*-promoter activity in the presence of the HDAC2 phosphor-mimic mutant HDAC2 S394E. As in Fig. 4a, PPP2CA significantly decreased *Nppa*-promoter activity. However, *Nppa*-promoter activity was fully recovered by expression of the phosphomimic mutant of HDAC2, S394E (Supplementary Figure 5).

**Fig. 6:**
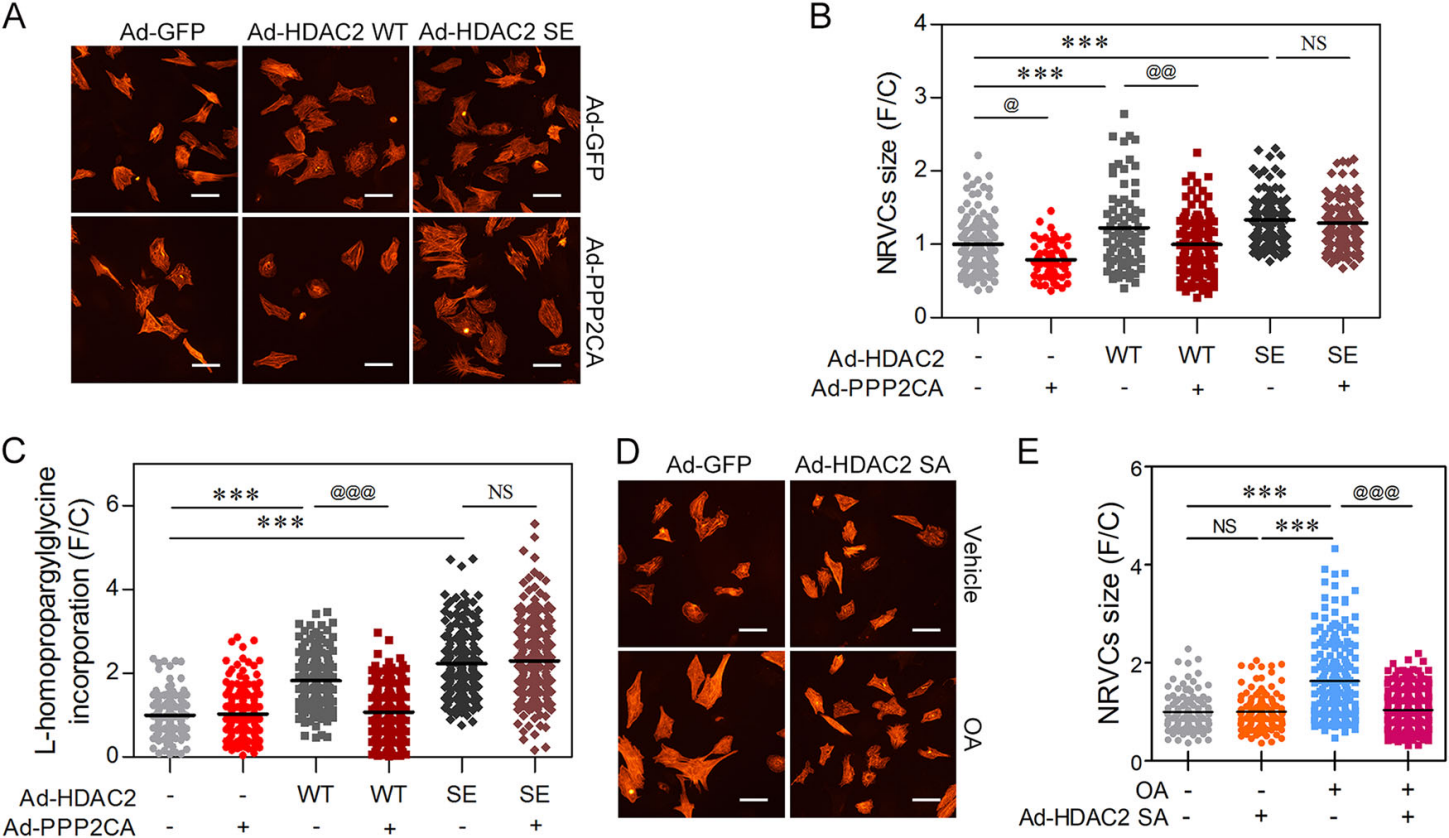
PPP2CA targets S394 phosphorylation of HDAC2 to modulate its hypertrophic signal. **a**, **b** Cell size measurements were performed in NRVCs infected with either Ad-HDAC2 wild-type (WT) or Ad-HDAC2 SE, phosphomimic mutant (SE indicates HDAC2 S394E). Both Ad-HDAC2 WT and Ad-HDAC2 SE successfully induced cardiomyocyte hypertrophy (**b**, 3rd and 5th group). Ad-PPP2CA abolished Ad-HDAC2 WT-driven cell enlargement. Ad-PPP2CA, however, failed to do so when Ad-HDAC2 SE was infected (**b**). **c** Quantification of HPG incorporation. NRVCs were infected with Ad-HDAC2 WT, SE, or Ad-PPP2CA for 48 h. Similar to 5 A and 5B, Ad-PPP2CA failed to suppress Ad-HDAC2 SE-driven cardiomyocyte hypertrophy. **d**, **e** OA-mediated hypertrophy was significantly attenuated by infection of Ad-HDAC2 SA, a phospho-resistant mutant of HDAC2 (SA indicates HDAC2 S394A). OA itself induced cardiomyocyte hypertrophy, which was significantly blunted when Ad-HDAC2 SA was infected. PPP2CA targets HDAC2 S394 phosphorylation for its negative regulation of cardiac hypertrophy. White solid bars depict 15 μm. @ indicates *p* < 0.05. @@ means *p* < 0.01. *** and @@@ indicate *p* < 0.001. NS not significant

In agreement with a previous report^16^, infection of NRVCs with Ad-HDAC2 WT resulted in cardiomyocyte hypertrophy (Fig. 6a, b 3rd column, Supplementary Figure 6A). Like the WT HDAC2, HDAC2 S394E, a constitutively active form of HDAC2 also induced cardiac hypertrophy (Fig. 6a, b 5th column, Supplementary Figure 6A). Interestingly, Ad-PPP2CA completely blocked HDAC2 WT-induced cardiomyocyte hypertrophy (Fig. 6a, b 4th column, Supplementary Figure 6A), whereas it failed to do so in HDAC2 S394E-expressing cardiomyocytes (Fig. 6a, b 6th column, Supplementary Figure 6A).

In addition to cell size measurements, we performed an HPG incorporation assay to provide evidence for the suggested mechanism of PPP2CA. Protein synthesis was dramatically increased either by infection of Ad-HDAC2 WT (Fig. 6c, 3rd column) or Ad-HDAC2 S394E (Fig. 6c, 5th column). As shown in Fig. 5b, Ad-HDAC2 S394E successfully increased HPG incorporation despite the infection of Ad-PPP2CA (Fig. 6c, 6th column), whereas Ad-HDAC2 WT failed to overcome the suppression of PPP2CA (Fig. 6c, 4th column).

Next, we postulated that OA-mediated cardiomyocyte hypertrophy was dependent on HDAC2 phosphorylation. To verify this hypothesis, we infected cells with a phospho-defective mutant of HDAC2, S394A, and examined the hypertrophic response. Ad-HDAC2 S394A blocked OA-induced cardiomyocyte enlargement (Fig. 6d, e, Supplementary Figure 6B). Taken together, our results suggested that PPP2CA reversed the phosphorylation of HDAC2 S394 and then abolished the cardiac hypertrophic phenotype.

### Regulation of the cardiac hypertrophy by PP2A via HDAC2 S394 dephosphorylation in vivo

**Fig. 7:**
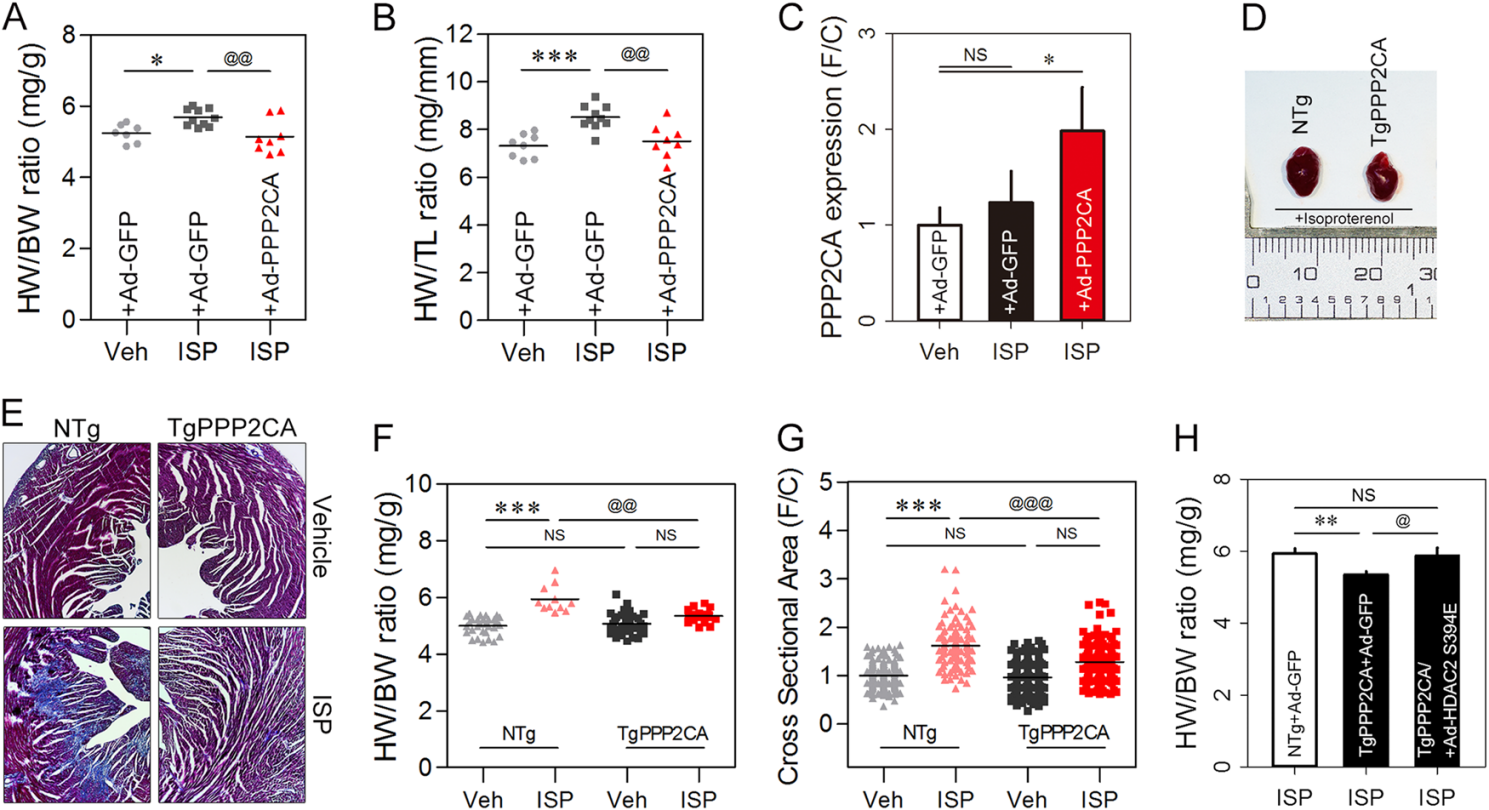
PPP2CA negatively regulates cardiac hypertrophy through HDAC2 dephosphorylation in the heart. **a**, **b** The anti-hypertrophic effect of PPP2CA was tested in CD1 mice. An in vivo hypertrophy model was tested using ISP infusion. Ad-PPP2CA was delivered via the tail vein 7 days after implantation of an ISP micro-osmotic pump, and the mice were sacrificed 14 days after the operation. Simultaneous expression of PPP2CA blunted ISP-induced cardiac hypertrophy, as determined either by heart weight per body weight ratio (HW/BW) (**a**) or by heart weight per tibia length ratio (HW/TL) (**b**). Dots depict individual mouse data. The mouse numbers used in **a** and **b** are as follows: 7 (Sham + Ad-GFP), 10 (ISP + Ad-GFP), and 8 (ISP + Ad-PPP2CA). **c** Quantitative real-time PCR revealed that Ad-PPP2CA successfully infected the myocardium. **d** Representative gross images. **e–g** Cardiac hypertrophy was significantly attenuated in TgPPP2CA mouse heart. Masson’s trichrome staining. Interstitial fibrosis induced by ISP infusion was dramatically reduced in TgPPP2CA mice (**e**). Transgenic expression of PPP2CA allowed resistance against the hypertrophy stimulus induced by infusion of ISP (**f**). Cross-sectional area in the left ventricle free wall showed a pattern similar to the HW/BW or HW/TL results (**g**). The anti-hypertrophic effect of PPP2CA against ISP infusion was not observed when Ad-HDAC2 S394E was expressed simultaneously in the heart of TgPPP2CA mice (H). * and @ depict *p* < 0.05. ** and @@ indicate *p* < 0.01. *** and @@@ mean *p* < 0.001. NS not significant

We next introduced an animal model to demonstrate the anti-hypertrophic effect of PPP2CA in whole heart. Ad-PPP2CA was introduced to the heart via tail vein injection in CD1 mice after implantation of a micro-osmotic pump that released ISP. Ad-PPP2CA infection blocked the ISP-induced cardiac hypertrophy, as measured by the heart weight to body weight (HW/BW) ratio or heart weight to tibia length (HW/TL) ratio (Fig. 7a, b). Expression of Ad-PPP2CA was confirmed by quantitative real-time PCR (Fig. 7c).

We next extended our observations in the animal model by utilizing transgenic mice. We evaluated cardiac hypertrophy and thereby fibrosis in mice expressing PPP2CA (TgPPP2CA). ISP induced a notable interstitial fibrosis, which was dramatically attenuated in the hearts of TgPPP2CA mice (Fig. 7e).

We did not find any evidence of a change in the HW/BW ratio in 7-week-old transgenic mice (Fig. 7f). Transgenic mice then underwent implantation of an ISP osmotic pump. ISP failed to induce cardiac hypertrophy in TgPPP2CA mice (Fig. 7d, f). Furthermore, we measured changes in the cross-sectional area of the left ventricle free wall and obtained a result similar to the hypertrophic phenotype after ISP implantation. Transgenic overexpression of PPP2CA significantly attenuated cardiac muscle enlargement (Fig. 7g). Next, we overexpressed HDAC2 S394E in the heart via tail vein injection of a virus. ISP failed to induce cardiac hypertrophy in the TgPPP2CA mouse heart, whereas significant cardiac hypertrophy was induced by adenoviral HDAC2 S394E. Taken together, our results suggest that PP2A acts as an endogenous phosphatase and an inhibitor of HDAC2 in the development of cardiac hypertrophy.

## Discussion

Cardiac hypertrophy is a muscle adaptation to external stress. Although hypertrophy itself is not a life-threatening disease, it is regarded as an intermediate stage that eventually turns into irreversible heart failure. Therefore, cardiac hypertrophy requires active intervention. Among hypertrophy-associated factors, the role of HDAC2 and its regulation have been relatively well established by our group. A series of studies have established HDAC2 as a crucial pro-hypertrophic regulator. The mechanism by which HDAC2 is regulated in hypertrophy, however, has not been fully described. In this study, we suggested a detailed mechanism for the regulation of HDAC2 phosphorylation, which is an essential modification to control HDAC2 activity.

**Fig. 8:**
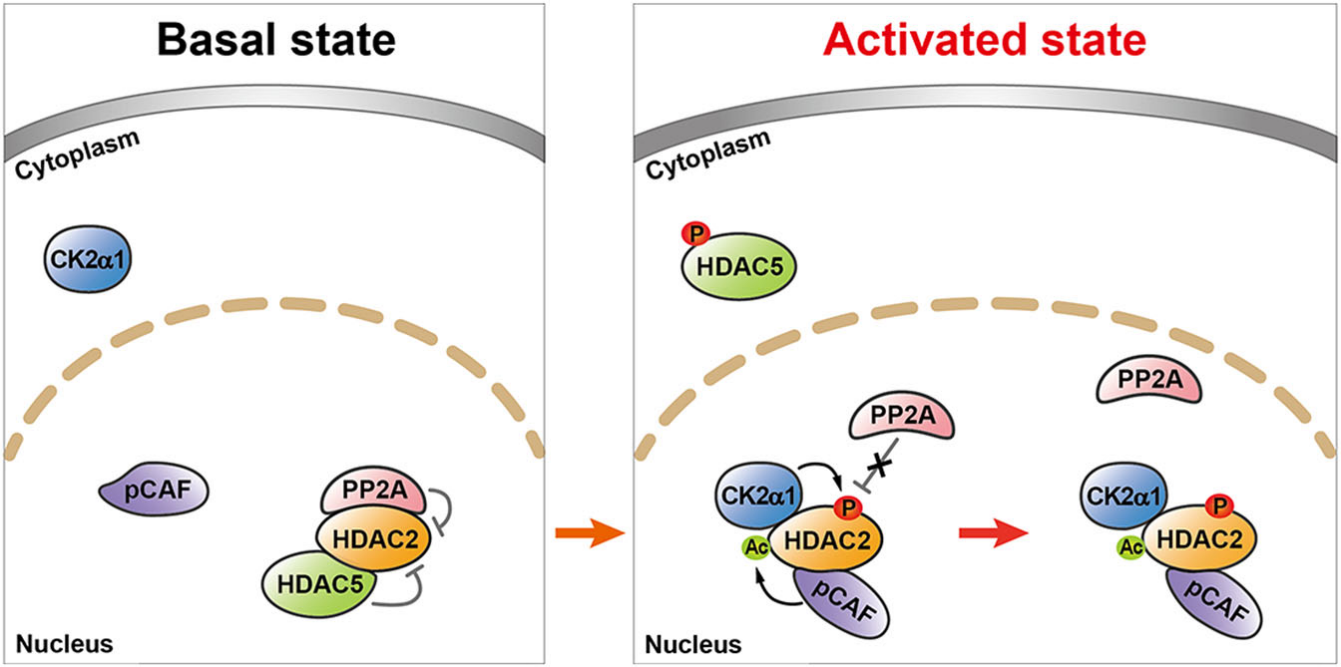
Working hypothesis. The diagram shows the working hypothesis based on the present work and the previously established mechanisms to regulate HDAC2 activity in the development of cardiac hypertrophy. HDAC2, a class-I HDAC^49^, and its posttranslational modification play an important role in the development of cardiac hypertrophy. In the basal state, HDAC2 is kept unphosphorylated and deacetylated by its association with PP2A and HDAC5, respectively (present work). In the activated state induced by hypertrophic stimuli, HDAC5 is then phosphorylated and shuttles out from the nucleus^13^. Simultaneously, pCAF interacts with HDAC2 to induce its acetylation^17^. After the acetylation of HDAC2, hypertrophic stresses further induce both the dissociation of PP2A (present work) and cytosol-to-nuclear relocalization of CK2α1^16^, which simultaneously result in the phosphorylation of HDAC2 S394. For ease of understanding, the hypertrophy non-responsive basal phosphorylation of HDAC2 is not included in the present diagram. Abbreviations: Ac, acetylation. P, phosphorylation

Synthesizing the present results with our previous findings, we suggest a novel working hypothesis, as depicted in Fig. 8. In the normal condition, both PPP2CA and HDAC5 tightly bind to HDAC2 and keep it inactivated. In response to hypertrophic stresses, CK2α1 is activated in the cytoplasm. HDAC5 phosphorylation occurs in the nucleus and HDAC5 is then shuttled to the cytoplasm. This process, in turn, gives rise to pCAF-dependent acetylation of HDAC2 in the absence of HDAC5, which then leads PPP2CA to dissociate from HDAC2. CK2α1 subsequently translocates into the nucleus and phosphorylates HDAC2.

Because HDAC2 is activated by a series of posttranslational modifications during the development of cardiac hypertrophy, the inactivation of HDAC2 could be used as a mechanism to inhibit cardiac hypertrophy. We have presented evidence of a negative regulation of PPP2CA that was transduced by transfection or by viral infection. Our findings are further supported by a recent study using cardiac-specific ablation of PPP2CA. Those investigators specifically introduced a tamoxifen-inducible deletion of PPP2CA in the heart and observed cardiac hypertrophy and its accompanying fibrosis 2 months after ablation of PPP2CA^32^. It is noteworthy that the chronic overexpression of PPP2CA, however, aggravated cardiac function and finally led to heart failure. Many independent research groups including ours^19,33–37^ have observed that heart failure is age-dependent in alpha-myosin heavy chain-driven TgPpp2ca mice; an enlarged chamber with decreased ejection fraction was observed at an age of over 3 months. The cardiac phenotypes are similar to that of dilated cardiomyopathy with eccentric hypertrophy. Unlike CK2α1, nuclear PPP2CA was not changed during the development of cardiac hypertrophy, which suggests that chronic overexpression of PPP2CA, as seen in TgPPP2CA, did not reflect the physiologic condition. Along with HDAC2, numerous other molecules have been reported as substrates of PP2CA, including c-Jun, Akt, ERK1/2, c-Myc, mTOR, and CDK1^38^. This led us to assume that the dilated cardiomyopathy phenotype is caused by the diversity of the targets of PPP2CA. Thus, although PPP2CA is an attractive pathway for inhibition of aberrantly increased HDAC activity, we should consider the notable side effects of chronic activation of PPP2CA.

As we have demonstrated in the present work, HDAC2 S394 phosphorylation is closely related to the development of diverse diseases, including inflammation^21^, obesity^39^, and oxidative stress-induced cell death^40^. As observed in other diseases, the inducible phosphorylation of HDAC2 S394, but not the basal phosphorylation at S422 and S424, is likely to be associated with pathophysiology. In particular, it is noteworthy that HDAC2 S394 phosphorylation and the subsequent increase in activity result in survival from oxidative stresses or tumorigenesis^40–43^. In the literature, various HDAC inhibitors have been highlighted as novel anticancer drugs. Several HDAC inhibitors have been approved to modulate the prognosis of hematologic malignancy, especially for cutaneous T-cell lymphoma^44^, and are in phase-II or phase-III clinical trials for solid tumors^45^. The final outcome of remission of solid tumors, however, was marginal^46^. The nonspecific inhibition of HDAC activity is likely to inhibit angiogenesis and results in the failure of further delivery of anticancer drugs, including the HDAC inhibitors themselves. To bypass the unwanted adverse effect of HDAC inhibitors, novel drugs that inhibit “disease-associated” activity should be developed.

Heart failure is defined as a decrease in cardiac function and a maladaptive step with resultant cardiac remodeling. The 5-year mortality for heart failure patients is greater than 50%, which is more severe than for several malignancies^47,48^. Once fatal remodeling starts, there are no appropriate remedies to reverse the pathology associated with the failing heart. Therefore, a therapeutic intervention to inhibit the transition to heart failure through HDAC inhibition and HDAC2 modulation, such as CK2 inhibition^16^ or activation of PPP2CA, may be a promising strategy for pathological cardiac hypertrophy.

## Electronic supplementary material

Supplementary Information
