# Author Correction: PP2A negatively regulates the hypertrophic response by dephosphorylating HDAC2 S394 in the heart

**DOI:** 10.1038/s12276-019-0222-6

**Published:** 2019-03-07

**Authors:** Somy Yoon, Taewon Kook, Hyun-Ki Min, Duk-Hwa Kwon, Young Kuk Cho, Mira Kim, Sera Shin, Hosouk Joung, Seung Hoon Jeong, Sumin Lee, Gaeun Kang, Yunchul Park, Yong Sook Kim, Youngkeun Ahn, Julie R. McMullen, Ulrich Gergs, Joachim Neumann, Kyung Keun Kim, Jungchul Kim, Kwang-Il Nam, Young-Kook Kim, Hyun Kook, Gwang Hyeon Eom

**Affiliations:** 1 Department of Pharmacology, Chonnam National University Medical School, Hwasun, 58128 Republic of Korea; 2 Medical Research Center for Gene Regulation, Chonnam National University Medical School, Hwasun, 58128 Republic of Korea; 3 Basic Research Laboratory for Cardiac Remodeling Research Laboratory, Chonnam National University Medical School, Hwasun, 58128 Republic of Korea; 4 Department of Pediatrics, Chonnam National University Hospital, Gwangju, 61469 Republic of Korea; 5 Division of Clinical Pharmacology, Chonnam National University Hospital, Gwangju, 61469 Republic of Korea; 6 Division of Trauma Surgery, Department of Surgery, Chonnam National University Hospital, Gwangju, 61469 Republic of Korea; 7 Biomedical Research Institute, Chonnam National University Hospital, Gwangju, 61469 Republic of Korea; 8 Department of Cardiology, Chonnam National University Hospital, Gwangju, 61469 Republic of Korea; 9 Baker Heart and Diabetes Institute, Melbourne, VIC 3004 Australia; 10 Institute of Pharmacology and Toxicology, Faculty of Medicine, Martin Luther University Halle-Wittenberg, 06097 Halle, Germany; 11 Department of Anatomy, Chonnam National University Medical School, Hwasun, 58128 Republic of Korea; 12 Department of Biochemistry, Chonnam National University Medical School, Hwasun, 58128 Republic of Korea

**Keywords:** Phosphorylation, Cardiac hypertrophy

**Correction to:** **Exp. Mol. Med.** (2018)50(7):83

10.1038/s12276-018-0121-2

After the publication of this article, the authors noticed an error in one of the grant numbers (2015R1A2A1A05001708) in the Acknowledgements section.

The correct statement of this article should have read as below.

“by a National Research Foundation of Korea grant funded by the Korean government (MEST, 2018R1A2B3001503).”

The authors apologize for any inconvenience caused.

