# Supplementary material for: PP2A negatively regulates the hypertrophic response by dephosphorylating HDAC2 S394 in the heart
Source: Exp Mol Med. 2018 Jul 26;50(7):83. doi: 10.1038/s12276-018-0121-2 (PMC6062565; doi:10.1038/s12276-018-0121-2)
Supplement: Supplementary file 1 — Supplementary Information [file 12276_2018_121_MOESM1_ESM.pdf]

SUPPLEMENTARY INFORMATIONS

Supplementary Figure Legends

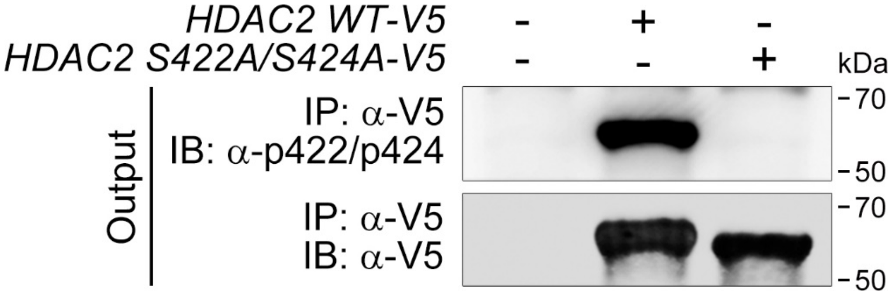

Supplementary Figure 1. Antisera against p422/p424 HDAC2 failed to recognize HDAC2 in which S422 and S424 were mutated to alanine.

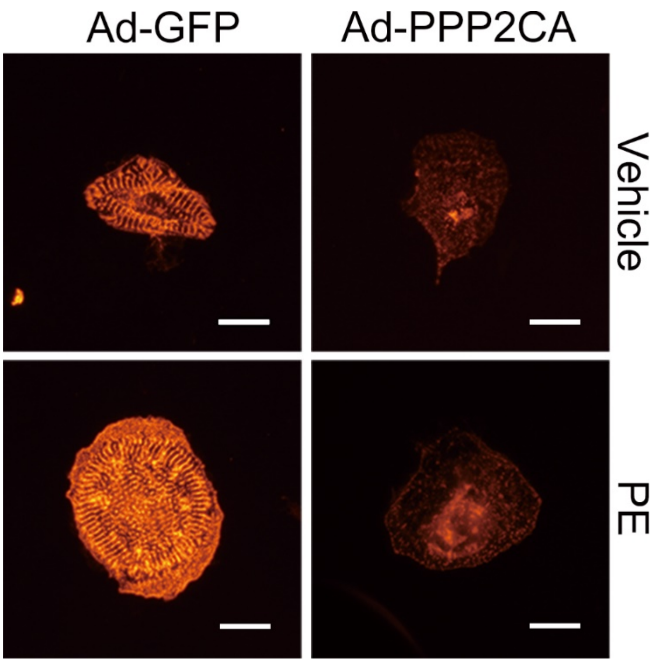

Supplementary Figure 2. High magnification images of individual cardiomyocyte in Figure 4B. PE induced both cell size enlargement and stress fiber formation, which was normalized by adenovirus expression PPP2CA. Scale bar means 15 μm.

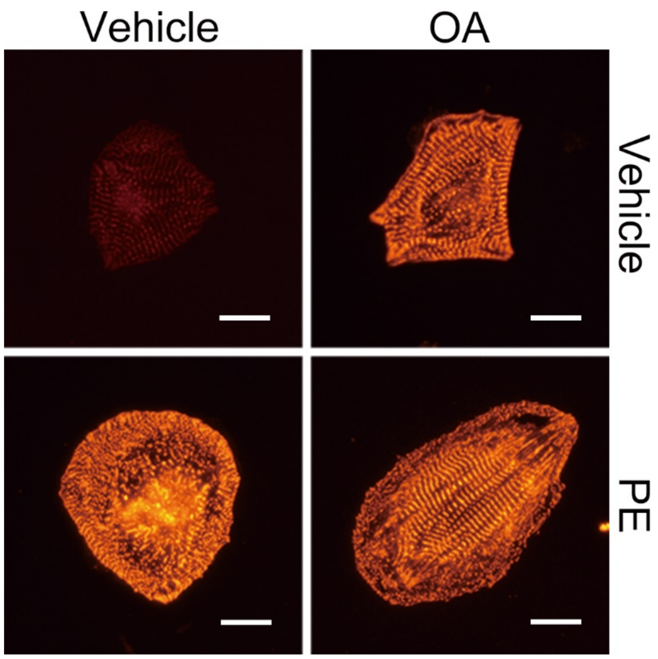

Supplementary Figure 3. High power field images of Figure 5A. PPP2CA inhibitor okadaic acid alone successfully induced cardiomyocyte hypertrophy and sarcomeric alpha actin formation as PE did. White bars indicate 15 μm.

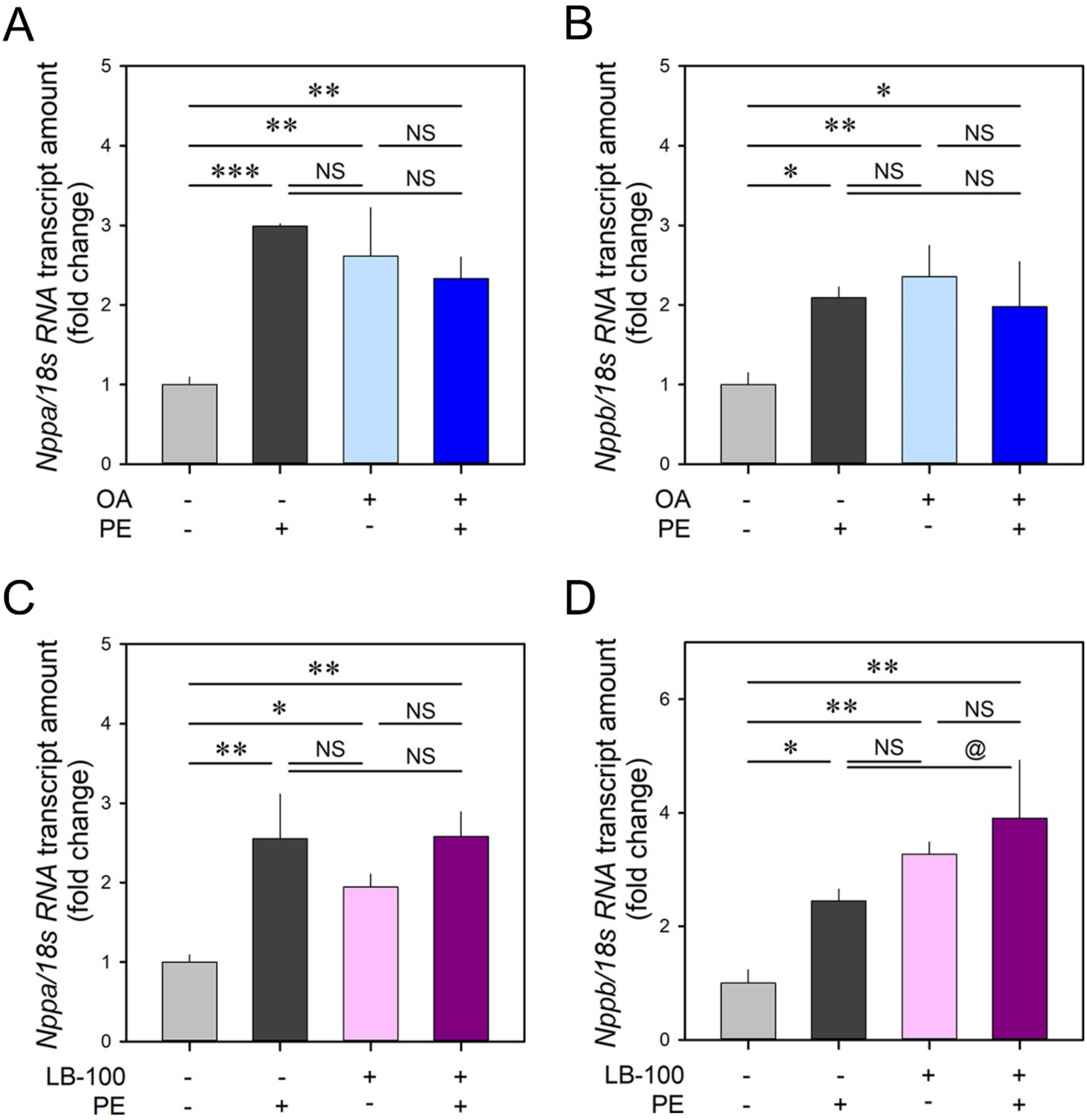

Supplementary Figure 4. PPP2CA inhibitors induced hypertrophic marker gene. Okadaic acid and LB-100 itself could induce *Nppa* and *Nppb* expression, but further potentiation by simultaneous treatment of PE with PP2A inhibitor was not observed.

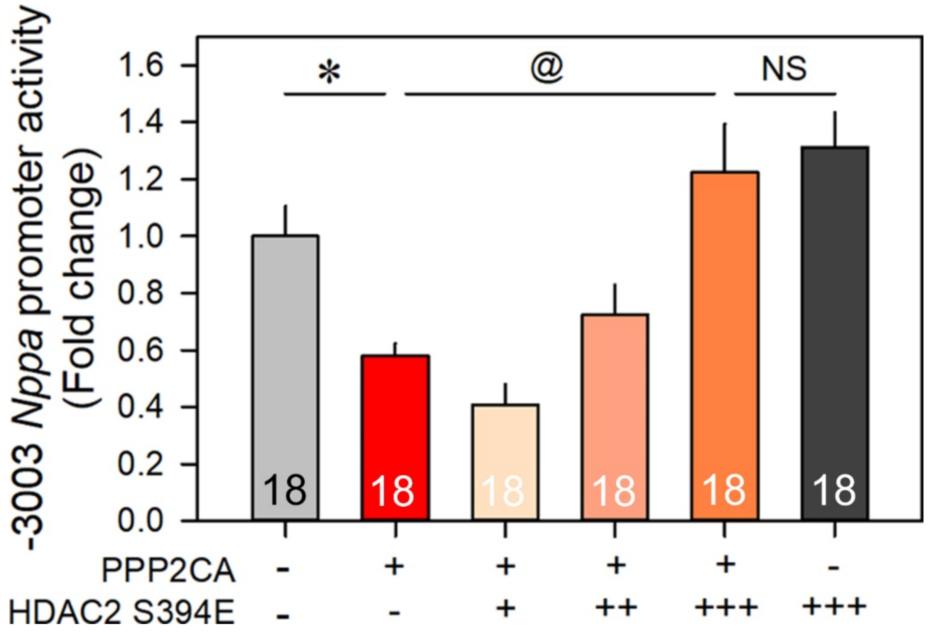

Supplementary Figure 5. PPP2CA targets S394 phosphorylation of HDAC2. Down-regulation of -3003 *Nppa*-promoter activity by PPP2CA was completely restored by forced expression of HDAC2 S394E, a phosphorylation-mimicking mutant of HDAC2. The white number in the bar graph indicates the number of independent experimental sets used for analysis. * and @ indicates $p<0.05$. ** means $p<0.01$. *** depicts $p<0.001$. NS means not significant.

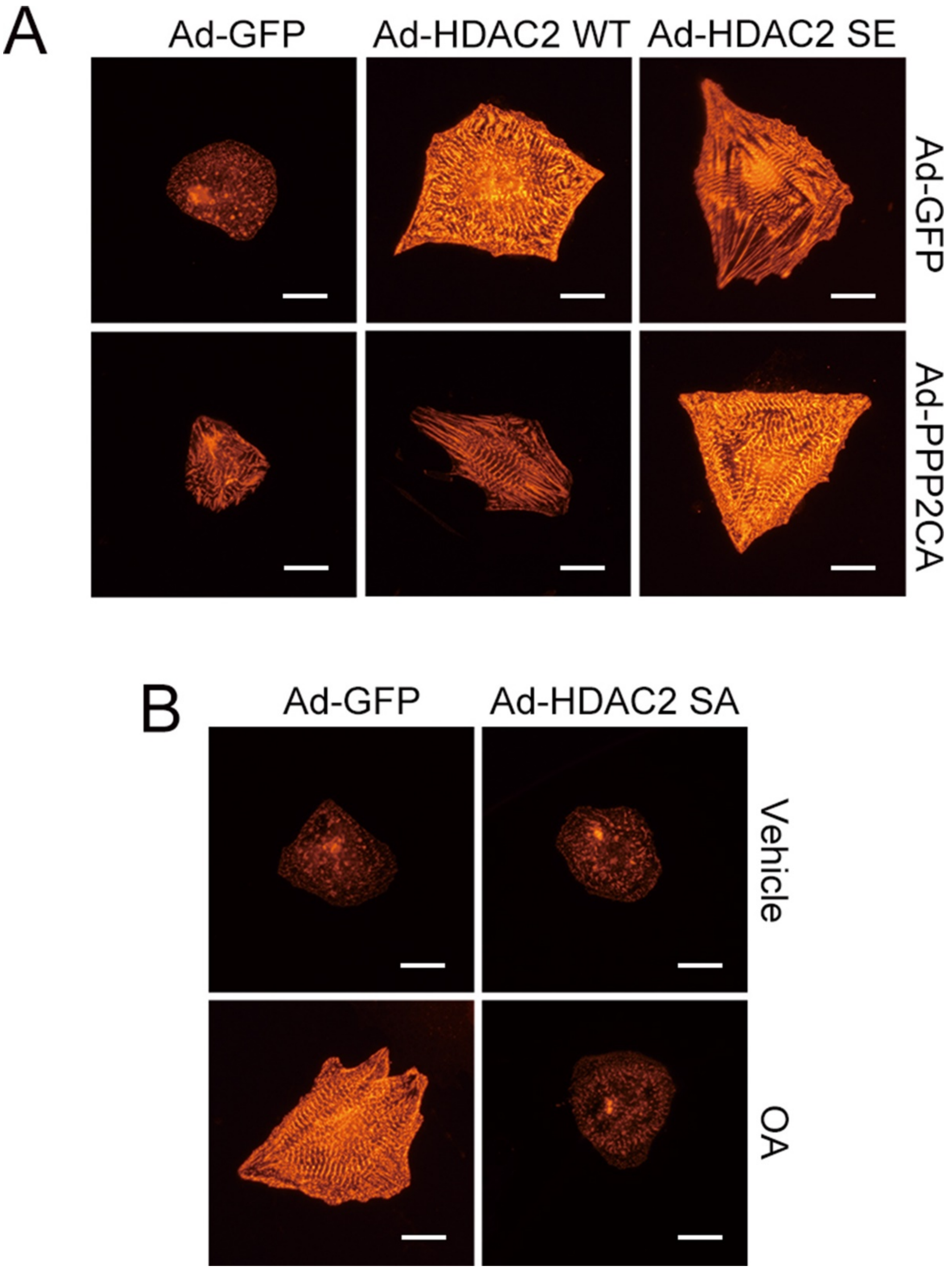

Supplementary Figure 6. High magnification images of Figure 6A and 6D. HDAC2 derived cardiomyocyte hypertrophy was completely reversed by Ad-PPP2CA, whereas infection of PPP2CA adenovirus failed to suppress hypertrophic response when phosphor-mimic HDAC2 (HDAC2 SE) adenovirus was infected (A). PP2A-inhibitor mediated hypertrophic phenotype was not detected when phosphor-dead mutant HDAC2 (HDAC2 SA) was expressed in the cardiomyocyte.
